# Identification of Factors Associated with Biofilm Formation Ability in the Clinical Isolates of *Helicobacter pylori*

**DOI:** 10.15171/ijb.1368

**Published:** 2017-03

**Authors:** Bahareh Attaran, Tahereh Falsafi

**Affiliations:** Department of Microbiology, Faculty of Biological Sciences, Alzahra University, Vanak, Tehran, Iran

**Keywords:** biofilm, Coccoid form, *Helicobacter pylori*, Mucins

## Abstract

**Background:**

A few reports confirm the ability of *Helicobacter pylori* to form biofilm. However, conclusive data do not exist concerning the factors that favor this ability.

**Objectives:**

Evaluation of the factors associated with the biofilm formation ability of *H. pylori* including bacterial, physical and chemical, and environmental factors was the research’s aim.

**Materials and Methods:**

*H. pylori* isolates from gastric biopsy specimens of patients infected chronically were screened for biofilm formation ability. Association of bacterial properties such as motility, auto-aggregation, cell hydrophobicity, and extracellular polymeric substances (EPS) with *in vitro* biofilm formation ability of *H. pylori* was evaluated. The effects of environmental factors such as growth-medium, temperature, oxygen-tension, pH, β-cyclodextrin, gastric secreted mucins, and sub-inhibitory concentration of amoxicillin were also evaluated.

**Results:**

Ability of clinical *H. pylori* isolates to form biofilm in was quantitatively compared. The coccoid shape *H. pylori* cells were observed by scanning electron microscopy, the images were illustrative of the attachment of cells to form microcolony. The levels of hydrophobicity, motility and auto aggregation of two isolates with highest and lowest biofilm formation ability were the same. However, the signifi cant role of mucins (P < 0.05) in elevating the biofilm formation was observed. Other factors influencing biofilm formation were: pH, atmosphere and sub-MIC of antibiotics.

**Conclusion:**

Mucins have a signifi cant role in elevating the biofilm formation, also pH, atmosphere and sub-MIC of antibiotics influence biofilm formation.

## 1. Background

Infection by *Helicobacter pylori* is associated with gastritis and peptic ulcer disease and may be a risk factor for gastric carcinoma and MALT lymphoma (Mucosa- associated lymphoid tissue) (1,2).

The biofilm mode of growth is a survival strategy deployed by many bacteria and is manifested as communities of cells attached to each other and/or to surfaces or interfaces, which are embedded in a self-produced matrix of extracellular polymeric substances (EPS) (3-5). Although biofilm formation would be slower *in vivo* than *in vitro*, once formed it may be able to induce the signaling process that conduct to the transcriptions of the bacterial genes, which are not expressed in planktonic cells. These signaling events may also be dependent on the nature of bacterial microenvironments, which can prevent or enhance its interaction with the host (6,7).

For a gastric pathogen such as *H. pylori* the host microenvironment would be very different from that of the exterior. After entry,* H. pylori* is surrounded by the host microenvironment, which contains mucins as integral part of the stomach mucosal barrier. Hence, the microenvironment surrounding the bacteria may also play a role in favoring or preventing production of the biofilm (8). The first report from the ability of *H. pylori* to form a biofilm indicated that this behavior may facilitate survival of bacteria in the stomach (9). Later studies indicated that bacterial biofilms are embedded in a self-produced extracellular matrix, which is a complex mixture of exopolysaccharides, proteins, DNA and other macromolecules (10). Furthermore, a polysaccharide-containing biofilm has been observed in the air-liquid interface on coverslips (7,10-12).

Presence of *H. pylori* under biofilm, has been observed in dental plaques or human gastric mucosa, as well as in the laboratories (1,12-17). However, the properties of* H. pylori* biofilm and the factors associated with its formation are not well studied.

## 2. Objectives

For a pathogen such as *H. pylori* the bacterial properties such as motility, auto-aggregation, cell hydrophobicity, and presence of the exopolymeric matrix of biofilms may be important in its survival and proliferation. Moreover, effects of some physical and chemical environmental factors such as temperature, pH, and aerobic or micoaerophil atmosphere or low concentrations of the antimicrobial agents are amongst the factors that *H. pylori* may encounter in its life cycle. For this purpose, these factors were evaluated by using of *H. pylori* isolates from chronic infection of children and adults, consisting of an efficient biofilm forming isolate and a weak biofilm forming isolate.

Identification of the effective factors involved in the biofilm formation by *H. pylori,* may help to better prevent its formation in host stomach. Furthermore, determination of the biofilm formation conditions, may help to select a better eradication regiments to circumvent biofilm formation and so chronic infection by antibiotic resistant bacteria.

## 2. Materials and Methods

### 2.1. Bacterial Isolates and Growth Conditions

A collection of 25 clinical *H. pylori* isolates from the chronic infection of children and adults were plated onto modified Campy blood agar containing brucella agar base (Merck, Germany), supplemented with 5% defibrinated sheep blood, and antibiotics (polymyxin B, amphotericin B, vancomycin), and incubated at 37°C under microaerobic atmosphere (10% CO_2_, 5% O_2_, and 85% N_2_) for three days. The grown colonies were identified by Gram staining, biochemical tests (catalase, oxidase, urease, nitrate) and PCR, using *H. pylori*-specific primers 16sRNA and ureC, as previously described (18).

### 2.2. Analysis of Biofilm by Staining

The grown colonies were harvested from culture plates and inoculated into brucella broth (Biolife, Italy) supplemented with 2% (w/v) fetal calf serum and 0.3% (w/v) glucose (Merck, Germany). Bacterial suspensions were incubated at 37°C in a microaerobic atmosphere under shaking at 100 rpm for 16 h. Broth cultures were adjusted to an optical density of 0.2 at 600 nm (A_600_) equivalent to a turbidity of 5-8 × 10^3^ colony-forming unit (CFU).mL^-1^, which corresponded to the beginning of exponential phase. Culture broth (250 μl) was inoculated into the polystyrene 96-well flat-bottomed tissue culture plates (BIOFIL, Jet Bio-Filtration Products Co., Ltd). The plates were incubated at 37°C under microaerobic condition for 6 days (19). The plates were vigorously washed (thrice) with sterile phosphate-buffered saline (PBS) (pH 7.2 - 7.4) in order to remove all non-adherent bacteria, were fixed with 99% ethanol for 20 min, air dried and stained with 1% Crystal Violet for 5 min; the excess of stain was rinsed away by running tap water and the dried plates were treated with 33% (v/v) glacial acetic acid, for solubilizing the attached dye. Optical density (OD) of the wells was measured at 505 nm according to the previously adopted protocol (20), using an ELISA reader (SCO, Germany).

### 2.3. Scanning Electron Microscopy of Biofilms

Cell suspensions were inoculated into 12-well cell culture plates. A coverslip was inserted into each well and the plate was incubated at 37°C under microaerobic conditions for 3 and 6 days. Thereafter, the coverslips were removed from the wells and rinsed off (by dipping in sterile medium) to remove non-adherent cells. They were fixed in 2% glutaraldehyde for 2 h and washed in PBS. The samples were dehydrated in a graded ethanol series of 25, 50, 75, 95, and 100% and stored in the desiccators until they were coated with gold-palladium sputter for two 200-second intervals (Nano Structured coating Co. Iran). SEM micrographs were performed using TESCAN VEGA3S electron microscope, at 30 KV.

### 2.4. Cell Viability Assay

Bacterial culture (2 mL) were seeded in 12-well culture plates (Nunc, Denmark), according to the protocol noted above, washed (thrice), and incubated with brucella broth, supplemented with 0.05% (w/v) of 2, 3, 5 triphenyl tetrazolium chloride (TTC) (Merck, Germany), at 37°C under microaerobic conditions. After 24 h, the broth was removed, the wells were air dried and the bound TTC dye was dissolved using 20% acetone -/80% ethanol; OD was then measured at 505 nm (21). To determine the number of biofilm forming bacteria, seeded culture plates were washed with sterile PBS (500 µL), the biofilm was removed with an ultrasonic bath for 7 min (Elmasonic S 60/ (H) - Germany, Ultrasonic frequency: 37 kHz), and CFU was determined. The results were presented as the mean of three independent tests (22).

### 2.5. Motility Assay

Motility of the *H. pylori* isolates were assessed by the method of Tan *et al*. (23). Bacteria were incubated in brucella broth supplemented with 3% serum was collected after 24 h, resuspended in PBS and the optical density (OD_600_) was adjusted to 1.0. Motility agar plates (0.35% agar medium containing brucella broth supplemented with serum), were inoculated with 5 µL of the bacterial suspension, and incubated under microaerobic condition at 37°C. The diameter of halos produced after 3-5 days were measured using three plates for each case.

### 2.6. Analysis of Biofilm Formation at the Air-Liquid Interface

A coverslip was placed into the wells of 12-well culture plates previously seeded with the bacterial cells as noted above, covered and incubated at 37°C under microaerobic conditions. After 3 and 6 days incubation, the coverslips were removed, rinsed off in sterile medium and the cells were stained by submersion in 0.1% crystal violet for 10 min. The biofilms were visualized by inverted microscope (24).

### 2.7. Cell Surface Hydrophobicity

Cell surface hydrophobicity was performed according to the method of Yonezawa and Osaki (11). Overnight bacterial culture (1 mL) in brucella broth was added to 9 mL of fresh medium and was incubated at 37°C for 20 h. Bacteria was harvested by centrifugation, washed (3´) with PUM buffer (0.15 mol.L^-1^ potassium phosphate buffer, pH 7.1, containing 0.3 mol.L^-1^ urea and 6.7 mmol.L^-1^ MgSO_4_), and resuspended in the same buffer. The resulting suspension was adjusted to OD_400_ 1.0, to which 600 mL of *n*-hexadecane (Merck, Germany) was added, and mixed vigorously for 60 s. After 15 min incubation at 22°C, the absorbance of the aqueous layer was measured at 400 nm. The cell surface hydrophobicity was expressed as follows:

(OD_400_ before mixing - OD_400_ after mixing) **/**OD_400_ before mixing × 100.

### 2.8. Auto-Aggregation Assay

Auto-aggregation assay was determined using a method adopted from Yonezawa *et al.* (11). Bacterial culture was washed, resuspended in PBS, adjusted to OD_600_ 1.0 and incubated at 22‏°C. ODs were read over time at 600 nm. The percent of auto-aggregation was measured as follows:

Auto-aggregation = (pre-incubation value [OD_600_] - incubation value [OD_600_])** /** (pre-incubation value [OD_600_] × 100.

### 2.9. Analysis of Extracellular Polymeric Substances (EPS)

Bacterial biofilms produced in 12-well cell culture plates (as noted above), were washed (thrice) with sterile distilled PBS and the cells were removed by incubation in an ultrasonic bath (Elmasonic S 60/ (H)-Germany, Ultrasonic frequency: 37 kHz) for 7 min. The cell suspension was extracted with 2% EDTA for 4 h at 4°C, centrifuged at 10000 ×*g*, 4°C for 20 min and the supernatants were filtered through a 0.22 µm polyvinylidene membrane (Fluoride, Millipore). The filtrates were used for determination of polysaccharide and protein contents according to the method adopted by Pan *et al.* (25). Polysaccharide content of EPS was determined by the phenolsulphuric acid method, according to Dubois and Gilles (26); glucose was used as the standard. Protein content of EPS was determined by the Bradford method (27) and the bovine serum albumin was used as the standard. In this analysis, the EPS of S7 clinical isolate of *Pseudomonas aeruginosa* was used as control (28).

### 2. 10. Effect of Mucins on Biofilm Formation

To evaluate the effect of native mucins (porcine mucins, Sigma Aldrich, USA) on biofilm formation, it was tested under various experimental conditions. First, various concentrations of mucins sterilized by gamma irradiation (dose of 3.5 kGy), was added to the culture medium using the previously described method (29), and the results of biofilm formation was compared. In the second case, the culture plates were coated with 1 mg/well of mucins (30), washed three times with PBS- 0.1% Tween-20, after overnight incubation at 37°C; the plates were then stored at 4-8°C until use. Quantitative detection of biofilm was performed by staining of the plates by crystal violet, Safranin also by TTC, followed by measurement of OD by ELISA reader.

### 2. 11. Evaluation of the Various Factors Affecting Biofilm Formation Using the Placket-Burman Design

Multiple factors that may affect the behavior of *H. pylori*, including growth-medium, addition of glucose, temperature, oxygen-tension, pH, b-cyclodextrin, mucins, and sub-inhibitory concentration of amoxicillin on the biofilm formation by *H. pylori* were studied according to placket-Burman design using Minitan16 software as demonstrated in Table 1.

**Table 1:** Placket-Burman design for study of 8 factors in 12 experiments.

**Std** **Order**	**Run Order**	**Pt Type**	**Blocks**	b**-cylodextrin** **%**	**Mucin** **g.L** ^-1^	**Amoxicillin** **g.mL** ^-1^	**Glucose** **g.L** ^-1^	**pH**	**Tempera-** **ture °C**	**Atmosphere**	**Medium**
1	1	1	1	0.02	0	0.002	0	2	20	aerophilic	brucella broth
2	2	1	1	0.02	0.005	0	0.02	2	20	microaerobic	brucella broth
3	3	1	1	0	0.005	0.002	0	7	20	microaerobic	f12
4	4	1	1	0.02	0	0.002	0.02	2	37	microaerobic	f12
5	5	1	1	0.02	0.005	0	0.02	7	20	aerophilic	f12
6	6	1	1	0.02	0.005	0.002	0	7	37	microaerobic	brucella broth
7	7	1	1	0	0.005	0.002	0.02	2	37	aerophilic	f12
8	8	1	1	0	0	0.002	0.02	7	20	aerophilic	brucella broth
9	9	1	1	0	0	0	0.02	7	37	microaerobic	brucella broth
10	10	1	1	0.02	0	0	0	7	37	aerophilic	f12
11	11	1	1	0	0.005	0	0	2	37	aerophilic	brucella broth
12	12	1	1	0	0	0	0	2	20	microaerobic	f12

The non-favorable temperature (28°C) was compared with 37°C; as well as the non-favorable atmosphere (aerophilic) was compared with microaerobic atmosphere; the very acidic (pH 2) was compared with pH 7. The effects of various media (brucella broth and F12) (31,32) in biofilm formation as well as the addition of glucose (2%) were evaluated (9). b-cylodextrin (12) and sub inhibitory concentration of amoxicillin were selected as the examples of the factors that may produce the favorable or non-favorable conditions for* H. pylori*. Each additional factor was added to the medium after sterilization (0.45 µm polyvinylidene fluoride, Millipore,). The role of mucins presence was also included in this design.

### 2. 12. Statistical Analysis

Statistical analysis was carried out using ANOVA one-way test with Minitab 16 statistical software, and probability levels of <0.05 were considered as statistically significant. For *in vitro* studies, three independent experiments with eight replicates were performed and in each test, the broth without bacteria was used as the negative control.

## 3. Results

### 3.1. In vitro Biofilm Formation by H. pylori Clinical Isolates

**Figure 1:**
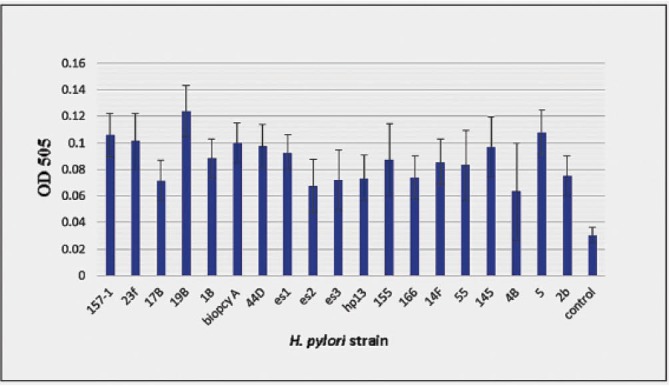
Quantitative measurement of biofilm formation for 19B using crystal violet staining after 6 days. All results are expressed as mean ± standard deviation from three independent experiments. Culture medium without bacteria use as a control.

Screening of the clinical isolates for biofilm forming ability showed that under favorable experimental conditions (brucella broth supplemented with 2% (w/v) fetal calf serum and 0.3% (w/v) glucose and incubation at 37°C in a microaerobic atmosphere with shaking at 100 rpm for 6 days) almost all of the isolates formed relatively little biomass on plates. Amongst which two isolates with significantly higher (19B) and lower (4B) levels of biofilm ability were selected (Fig. 1). 19B was isolated from an 11years-old female patient with endoscopic status of severe gastritis and pathology of moderate active chronic gastritis. 4B was isolated from a 38 years-old male patient with endoscopic status of normal and pathology of mild chronic gastritis.

### 3.2. Scanning Electron Microscopy of Biofilm

**Figure 2:**
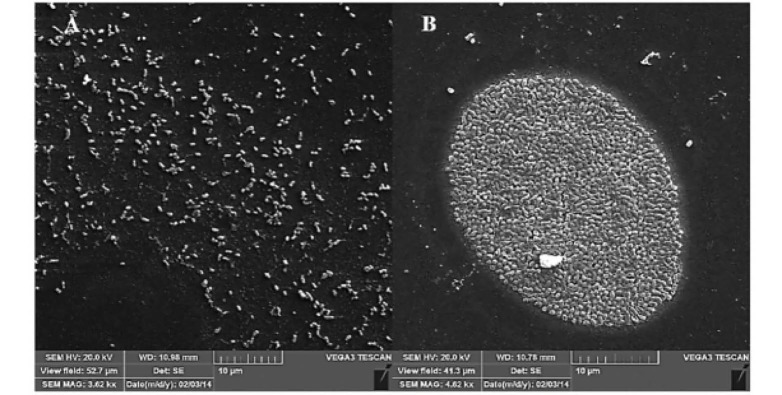
Scanning electron microscope of biofilm formation by *H. pylori* 19B. A: cells recovered from supernatant of 6-days growth (planktonic populations). B: biofilm at day 6 after growth.

SEM micrograph of biofilm forming cells after 6 days (Fig. 2) demonstrated that a tight attachment of coccoid bacterial cells was formed in microcolonies. This morphology could be compared with that of the planktonic populations recovered from the supernatant of the same isolate.

### 3.3. Cell Viability Assay

Viability of the biofilm forming cells was evaluated after 3-day and 6-day by two methods of viable counting (CFU) and straining method using Crystal Violet as well as TTC. Comparison of the staining results for two periods demonstrated that OD of TTC-stained cells were almost similar (Table 2). Similarly, the number of bacteria counted by CFU method was also comparable for two periods.

**Table 2:** Comparison of CFU enumeration with the results of Crystal violet and TTC staining for biofilms of *H. pylori* isolates 19B and 4B after 3 and 6 days.

*** H. pylori strain ***	** Days after incubation **	** CFU/well ± SD **	** OD of Crystal violet stained cells at 505 nm ± SD **	** OD of TTC stained cells at 505 nm ± SD **
19B	3 days	0.388 ± 0.154 × 10^ 9^	0.111 ± 0.009	2.225 ± 0.342
4B	3 days	0.522 ± 0.166 × 10^5^	0.066 ± 0.007	0.640 ± 0.078
19B	6 days	0.403 ± 0.112 × 10 ^9^	0.136 ± 0.017	2.125 ± 0.120
4B	6 days	0.594 ± 0.139 × 10 ^5^	0.069± 0.0136	0.674 ± 0.071
Control	-	0	0.0318 ± 0.005	0.0571 ± 0.069

### 3.4. Air-Liquid Interface Coverslip (CV) Assay

Both isolates formed a biofilm at air-liquid interface on coverslip surface. However, 19B formed denser community on the glass surface compared to 4B.

### 3.5. Motility, Hydrophobicity, and Auto-Aggregation Assays

**Figure 3:**
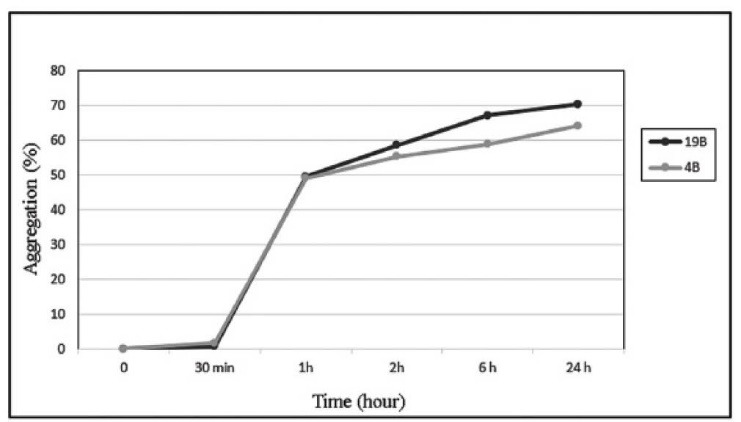
Auto-aggregation assay for *H. pylori* isolates 19B and 4B. The percent of auto-aggregation was calculated and the results were expressed as the mean of three independent experiments.

19B and 4B were both motile with no significant differences between their motility halos. Their hydrophobicity levels were also similar (63.7 ± 0.452 for 19B and 68.36 ± 1.108 for 4B). Concerning their auto-aggregation abilities, both isolates showed strong auto-aggregation ability with no significant difference (Fig. 3).

### 3.6. Evaluation of Polysaccharide and Protein Contents in EPS

By comparison of protein and polysaccharide content in the EPS of *H. pylori* isolates, 19B and 4B, expressed in their percentage (relative to the total weight of the biofilm), no significant differences were observed between the two. However, their percentages were significantly higher in *Pseudomonas aeruginosa* S7, in comparison with 19B and 4B.

### 3.7. Effect of Mucins on Biofilm Formation

**Figure 4:**
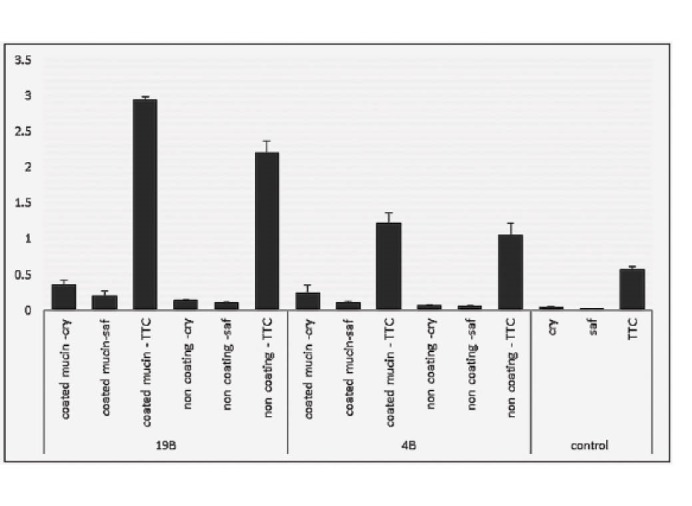
Effect of mucins on biofilm formation of *H. pylori* isolates 19B and 4B. For each sample three staining method was evaluated. TTC staining for viability assessment and OD_595_ was measured. Crystal violet (cry) and Safranin (saf) assessed and OD_505_ was measured.

Addition of various concentrations of mucins to the culture medium, had no significant effect on the biofilm formation ability; but coating of plates with mucins before bacterial loading, demonstrated a significant positive effect on the biofilm formation by *H. pylori* (Fig. 4).

### 3.8. Placket-Burman Design

**Figure 5:**
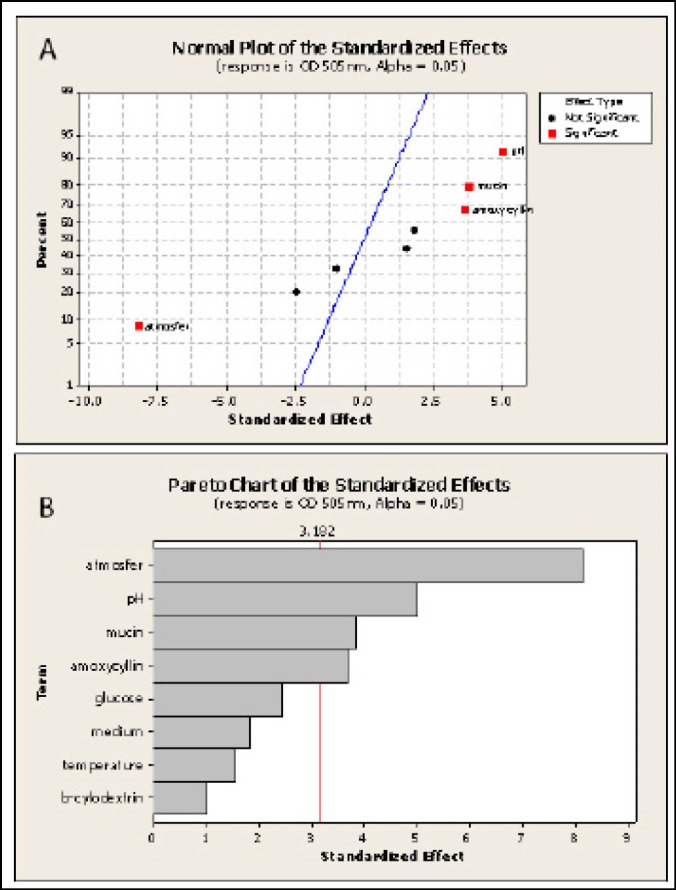
Normal plot and Pareto Chart of the effect of growth medium, temperature, mucins, pH, sub MIC concentration of amoxicillin and oxygen tension on *H. pylori* isolate 19B biofilm formation evaluated by crystal violet staining. (A) Normal plot of factors, Using ANOVA following factors: atmosphere (oxygen tension), pH, mucins and sub MIC amoxicillin are significant to the response. (B) pareto chat of standard effects.

The effects of growth medium, addition of glucose to the growth medium, temperature, pH, sub-MIC concentration of amoxicillin, b-cyclodextrin, various incubation atmosphere (oxygen tension), and presence of mucins, on *H. Pylori* biofilm formation is demonstrated in Figure 5. Among various favorable factors atmosphere (microaerobic), pH 7.0 , presence of mucins and sub-MIC concentration of amoxicillin had significant effects (*p* < 0.005) on biofilm formation by *H. pylori*.

## 4. Discussion

Significance of effective factors in biofilm formation of* H. pylori* has not been well evaluated. By selecting a pair of strains consisting of an efficient biofilm-forming and a weak biofilm-forming, important factors were evaluated. Through understanding the factors that affecting biofilm formation by this pathogen, one may encounter the *H*. *pylori* and stop the biofilm formation in the future.

Initial evaluation of biofilm formation by *H. Pylori* clinical isolates were performed by crystal violet staining method. For quantitative evaluation of biofilm formation and count of viable cells, an optimized TTC staining method was used (21). Its advantage is that metabolically active bacteria reduce 2, 3, 5 triphenyltetrazolium chloride (TTC) to insoluble, red crystals of 1, 3, 5-triphenylformazan (TFP), which can be measured calorimetrically. TTC data were more consistent with those of CFU (Table 2). It seems that TTC staining is an accurate method to evaluate both biofilm formation by *H. pylori* and its viability.

Assessment of biofilm formation by electron microscopy in 19B and its planktonic populations showed tight adherence between biofilm forming cells, which lead to the microcolony formation (Fig. 2). Presence of coccoid form cells, which were firmly attached together, suggests that this state may be dominant in the *H. pylori* biofilm. This observation is consistent with other *in vitro* studies demonstrating that coccoid form *H. pylori* is present more in its biofilm state (7). Consistent with an earlier report (12),* H. pylori* isolates used in this work were able to form a biofilm at air-liquid interface on coverslip surface. However, the biofilm of 19B demonstrated the dense communities attached to the glass surface.

Contrary to the results of Yonezawa *et al*. (11), no significant differences were observed between the hydrophobicity levels, motility and auto-aggregation of 19B and 4B. This difference in the results may be related to the differences among the isolates employed in the two studies.

Potentially, the nature of EPS and its amount may play a role in biofilm formation (25). Although resistance of biofilm forming cells to antibiotics may be a very complex phenomenon, bioﬁlm forming cells may be protected by their EPS via prevention of antimicrobial penetration (34).

In the case of *H. pylori*, only one report has evaluated its EPS, and demonstrated 1, 4-mannosyl linkages in *H. pylori* biofilms and mannose as main sugar (35). We assessed the amount of polysaccharide and protein in EPS of *H. pylori* isolates and compared their contents with those of *Pseudomonas aeruginosa* S7 isolates (Table 3). Although their contents were lower than those of *P. aeruginosa* biofilms, their significant amounts indicated that such as the case of *P. aeroginosa*, the polysaccharide and protein content of EPS may be important for *H. pylori*.

**Table 3:** Protein and polysaccharide contents of biofilm EPS in biofilm of *H. pylori* isolates 19B and 4B compared with those of *P. aeroginosa*. The data are representative of 3 independent experiments.

*** H. pylori*** ** isolates**	** Polysaccharide (μg)/Well**	** %Polysaccharide /Biofilm wet weight**	** Protein (μg)/well**	** % Protein /biofilm wet weight**
19B	12.31 ± 4.57	25.65 ± 9.53	16.29 ± 1.77	33.93 ± 3.70
4B	8.69 ± 0.77	31.05 ± 2.76	11.77 ± 0.55	42.04 ± 1.98
S7 P. aeruginosa	44.09 ± 6.97	49.54 ± 7.83	27.05 ± 1.56	30.39 ± 1.76

The only one study analyzing the role of mucins in biofilm formation by *H. pylori* reports no effect on the biofilm formation or on the number of adherent bacteria (12). Contrary to this finding, our result showed that mucins significantly increase biofilm formation by *H. pylori* (Figs. 4 and 5). The effect of mucins on the adherence of bacteria to culture plates as well as on the number of biofilm forming bacteria were analyzed. On the contrary to their method of sterilization which was by autoclaving that cause structure damage, we conserved the native structure of mucins by gamma irradiation using a dose of 3.5 kGy, which was proven to maintain its integrity (29).

To determine effective factors in biofilm formation by this pathogen, Placket-Burman design was used. Accordingly, the positive role of four factors were demonstrated. The factors were microaerobic atmosphere, pH (pH 7), mucins and sub-MIC concentration of amoxicillin, as the favoring factors in the biofilm formation (Fig. 5).

Presence of mucins or microaerobic conditions as well as sub MIC concentration of the antibiotic (due to inappropriate doses of antibiotics), may be amongst the factors that *H. pylori* may encounter in its life cycle. Also in natural conditions of stomach, *H. pylori* may prefer neutral condition to acidic pH for biofilm formation.

Reuter and *et al*. (36) have shown that biofilm formation by *Campylobacter jejuni* is enhanced under aerobic condition perhaps due to the fact that *C. jejuni* could adapt to the aerobic condition and survived in this atmosphere via biofilm formation. Although this adaptation to the environment may be important for survival during transmission (36), we observed that *H. pylori* was not able to act similar to *C. jejuni*, perhaps due to its slower growth rate in aerobic conditions or its more sensitivity to oxygen pressure. A decrease in biofilm formation at pH 2 was observed that suggests that the presence of an active urease is not solely sufficient to circumvent the low pH. Since *H. pylori* uses its flagella to reach the neutral environment of the gastric epithelial cells of distal stomach. Consistent with this suggestion, the non-motile mutants of *H. pylori* are less virulent. Furthermore, freshly obtained isolates from stomach had the spiral morphology, whereas those subcultured in the laboratory had bacilli or even coccoid morphology. Motility of the two isolates with high and low biofilm forming ability were almost similar suggesting that for *in vitro* biofilm formation, *H. pylori* does not require motility.

Using the sub MIC concentration of amoxicillin, an increase in biofilm formation was observed. By creation of non-favorable conditions for bacteria, the sub-MIC concentration of amoxicillin favors biofilm formation, which helps bacteria better resist this drug. Bessa *et al*. (32) have reported that sub-MICs of amoxicillin and clarithromycin increased biofilm formation in *H. pylori*. This kind of resistance helps bacteria circumvent a stress condition or resist a drug, a situation that may be occurred in stomach since a biofilm formed *in vivo* may be a real barrier to the antibiotics. Clarification of this resistance in *H. pylori* may help to develop more effective treatment regimens as well as strategies other than the traditional means (37).

By screening of the clinical* H. pylori*, we demonstrated their ability to form a typical bacterial biofilm *in vitro*. Also, by evaluating the role of various environmental factors, we could demonstrate the favorable factors that enhance the biofilm formation ability *in vitro*. They include presence of mucins, favorable pH and microaerophil atmosphere and sub-MIC concentration of antibiotics, the roles which may be extrapolated to *in-vivo* conditions.

The results of present study that suggest favorable roles of multiple factors in biofilm formation, may also be important in biofilm formation by *H. pylori* in stomach*.* Evaluation of the factors that increase biofilm formation by *H. pylori* in animal models may be useful in planning of the biofilm formation prevention and as a result reducing generation of antibiotic resistance and chronic infection.

## Acknowledgments

We cordially thank Dr. Sara Gharavi from Alzahra University, Tehran, Iran, for editing of this article.
